# Genomic targets for high-resolution inference of kinship, ancestry and disease susceptibility in orang-utans (genus: *Pongo*)

**DOI:** 10.1186/s12864-020-07278-3

**Published:** 2020-12-07

**Authors:** Graham L. Banes, Emily D. Fountain, Alyssa Karklus, Hao-Ming Huang, Nian-Hong Jang-Liaw, Daniel L. Burgess, Jennifer Wendt, Cynthia Moehlenkamp, George F. Mayhew

**Affiliations:** 1 Wisconsin National Primate Research Center, University of Wisconsin–Madison, 1220 Capitol Court, Madison, WI 53715 USA; 2 School of Veterinary Medicine, University of Wisconsin–Madison, 2015 Linden Drive, Madison, WI 53706 USA; 3 Conservation Genetics Laboratory, Conservation and Research Center, Taipei Zoo, No. 30, Section 2, Xinguang Road, Wenshan District, Taipei City, Taiwan 11656; 4 Roche Sequencing Solutions, 500 S Rosa Road, Madison, WI 53719 USA; 5 Polymer Forge, Inc., 504 S Rosa Rd Ste 200, Madison, WI 53719 USA; 6 Promega Corporation, 2800 Woods Hollow Rd, Fitchburg, WI 53711 USA; 7 Exact Sciences, 441 Charmany Dr, Madison, WI 53719 USA

**Keywords:** Ancestry informative markers, Cardiac disease, Chronic respiratory disease, Pedigree reconstruction, Baits, In-solution capture, ACMG v2.0

## Abstract

**Background:**

Orang-utans comprise three critically endangered species endemic to the islands of Borneo and Sumatra. Though whole-genome sequencing has recently accelerated our understanding of their evolutionary history, the costs of implementing routine genome screening and diagnostics remain prohibitive. Capitalizing on a tri-fold locus discovery approach, combining data from published whole-genome sequences, novel whole-exome sequencing, and microarray-derived genotype data, we aimed to develop a highly informative gene-focused panel of targets that can be used to address a broad range of research questions.

**Results:**

We identified and present genomic co-ordinates for 175,186 SNPs and 2315 Y-chromosomal targets, plus 185 genes either known or presumed to be pathogenic in cardiovascular (*N* = 109) or respiratory (*N* = 43) diseases in humans – the primary and secondary causes of captive orang-utan mortality – or a majority of other human diseases (*N* = 33). As proof of concept, we designed and synthesized ‘SeqCap’ hybrid capture probes for these targets, demonstrating cost-effective target enrichment and reduced-representation sequencing.

**Conclusions:**

Our targets are of broad utility in studies of orang-utan ancestry, admixture and disease susceptibility and aetiology, and thus are of value in addressing questions key to the survival of these species. To facilitate comparative analyses, these targets could now be standardized for future orang-utan population genomic studies. The targets are broadly compatible with commercial target enrichment platforms and can be utilized as published here to synthesize applicable probes.

**Supplementary Information:**

The online version contains supplementary material available at 10.1186/s12864-020-07278-3.

## Background

Advances in analytic molecular methods have gradually shed light on the evolutionary history of orang-utans (*Pongo* spp.). Protein electrophoretic studies, beginning in the 1970s [1, 2], first supported the description of two subspecies, distinct to the islands of Borneo and Sumatra. Each was upgraded to species in 2000, following complete mitochondrial genome sequencing [3], and Bornean orang-utans were split into subspecies in 2003, based largely on further mitochondrial data [4, 5]. The first orang-utan reference genome was generated in 2011 [6], before the genus was split into three species in 2017, following whole genome re-sequencing of a previously understudied population [7]. Today, three species are formally recognized on the islands of Sumatra (*Pongo abelii; P. tapanuliensis*) and Borneo (*P. pygmaeus*). The latter is still divided into three subspecies in the western *(P. p. pygmaeus)*, central *(P. p. wurmbii)* and eastern *(P. p. morio)* regions of the island [4, 5].

Our understanding of orang-utan taxonomy and evolution has fast outpaced their survival. More than 100,000 Bornean orang-utans were reportedly killed in the wild from 1999 to 2015, 50% of which were lost from forests affected by natural resource extraction [8]. All three species are now critically endangered: fewer than ~ 57,000 reportedly survive on Borneo, while ~ 13,800 Sumatran and ~ 800 Tapanuli orang-utans are thought to remain on Sumatra [9]. Consequently, surviving wild orang-utans are increasingly intensively managed by humans, whether intended or not. Long runs of homozygosity have been observed in the genomes of wild Tapanuli orang-utans, suggesting inbreeding is occurring due to anthropogenic range restriction [7]. On Borneo, orang-utans of non-native subspecies are known to have been translocated and unwittingly returned to the wild, despite diverging ~ 176,000 years ago, and being subject to marked genetic differentiation over the last ~ 82,000 years [10]. Meanwhile, ~ 1500 orang-utans are still awaiting reintroduction from rehabilitation centres in-situ. There is no legal requirement to genetically test these individuals and return them to their regions of origin, despite there being no understanding of the effects of such admixture. Though the potential for outbreeding depression has been cited, orang-utans’ large home ranges and long generation times render it impractical to investigate its incidence in the wild [11].

In contrast, ex-situ orang-utans in zoos might serve as model populations for studying the effects of human intervention. Approximately 1100 orang-utans live in zoos worldwide, although numbers are probably higher in developing nations and in range countries [12]. Zoo populations of orang-utans are known to be highly admixed. Until the 1990s, Bornean and Sumatran orang-utans were inter-bred in zoos, producing a hybrid population that has since been contracepted. The extent to which the Tapanuli species is represented in zoos is unclear. Beyond the species level, captive Sumatran orang-utans have been shown to be highly admixed among those from distinct geographic subpopulations, while those of Bornean origin are known to have introgressed among all three subspecies. These hybridizations have occurred rapidly over multiple generations, given the far shorter inter-birth intervals than would naturally occur in the wild [13]. It is notable that significant health conditions are increasingly prevalent in zoo populations, with cardiovascular and chronic respiratory diseases comprising the primary and secondary causes of mortality. The former caused 16% of adult deaths in US zoos and was reported in up to 40% of living animals; 28.9% of all sub-adult and adult deaths were attributed to the latter, which was otherwise a contributing factor in 12% of all other deaths [14, 15]. As neither has been confirmed in wholly natural populations, each is assumed to be the product of intensive genetic or environmental management [16, 17].

As we consider how best to manage displaced orang-utans [11, 18], and how best to secure a sustainable future for those in zoos (sensu [19]), the need to better understand their genetic diversity – and the implications of their admixture – is becoming increasingly pressing. To date, most studies have utilized microsatellites to infer admixture and kinship, relying on non-invasive (i.e. faecal, hair) sampling techniques [10, 20–29]. These studies lack the resolutions necessary to build distant pedigrees, however, and – as so many orang-utans are now unnaturally admixed, both in ex-situ and reintroduced populations – their methods use too few loci to infer complex hybridization [30]. Oppositely, whole-genome sequencing approaches are cost-prohibitive on a large scale, in terms of both laboratory and computational costs; hence, only 38 individual genomes have been (re-)sequenced to date [6, 7, 31]. At high coverage, whole-genome sequencing also typically requires high quantities of high-molecular-weight DNA, as do microarray studies: in both cases, at least hundreds of nanograms. Samples of this quality are usually only available from captive individuals, and under strict legal and institutional requirements for animal care and use.

Here, we present a panel of molecular targets that can facilitate standardized comparative studies of orang-utan genomic variation. We adopt a reduced-representation sequencing approach, which can be used to consistently target loci of specific interest in high numbers and at high coverage, from lower input quantities of genomic DNA (i.e. *≤* 100 ng). Our panel can be used to infer ancestry and kinship at high resolutions; trace origins and assess admixture in sampled populations; and as a platform for investigating chronic respiratory and cardiovascular disease susceptibility and aetiology. These markers are of broad utility in studies that seek to better understand orang-utan evolutionary biology and health.

## Methods

### Selection of ancestry- and kinship-informative SNPs

We mapped published sequence reads from 37 whole genomes, derived from three prior studies [6, 7, 31], to the latest iteration of the orang-utan reference genome (ponAbe3, [32]) (Table 1). We used the Burrows-Wheeler Aligner (BWA-MEM) 0.7.17 [33] and samtools 1.9 to produce a BAM file [34], and Picard 2.20.2 to assign read groups and filter duplicates [35]. We then called variants using the GATK 4.1.8.0 (specific tools noted in parentheses) [36], broadly following the Best Practice workflows with modifications for non-human data [37]. Thus, we first performed initial rounds of haplotype calling (HaplotypeCaller), imported and genotyped the haplotypes from a GenomicsDB (GenomicsDBImport, GenotypeGVCFs), and selected and hard-filtered the outputs using the following parameters: QD < 2.0, MQ < 40.0, FS > 60.0, SOR > 3.0, MQRankSum < − 12.5, ReadPosRankSum < − 8.0 for SNPs; QD < 2.0, ReadPosRankSum < − 20.0, InbreedingCoeff < − 0.8, FS > 200.0, SOR > 10.0 for INDELs (SelectVariants; VariantFiltration). To correct for systematic sequencing errors, we used the hard-filtered outputs to perform empirical base quality score recalibration (BQSR; BaseRecalibrator), repeating the entire process until convergence (in practice, twice). We repeated all these steps, up to BQSR, on the recalibrated BAM files. To perform variant quality score recalibration (VQSR; VariantRecalibrator), we used the hard-filtered SNPs as a training set, plus 250,000 microarray-derived SNPs as a truthing set (see below), with a truth sensitivity filter of 99.8%. To discover low-frequency alleles across the genus, we applied the workflow four times: first, comprising all genomes, and subsequently, comprising genomes from each orang-utan species separately. Having parallelized the workflow across genomic intervals, we combined all intervals per species (GatherVcfs), before merging all sites (without genotypes) from the final Bornean, Sumatran, Tapanuli and Genus VCF files into a master set of high-confidence loci (MakeSitesOnlyVcf; GatherVcfs,). Capitalizing on the new –include-non-variant-sites flag in the GATK 4.1.2.0, we then re-called haplotypes and re-genotyped all samples, using the master loci set as an interval list. This facilitated consistent genotyping of all loci across all samples, with no missing data. All computational analyses were performed via HTCondor [38]; data were distributed via StashCache [39].

**Table 1:** Published, re-sequenced genomes from 37 orang-utans were used in panel development

No.	ID	Sex	Citation	Origin
1	356	F	[6]	Bornean *(Pongo pygmaeus)*
2	360	M		
3	364	F		
4	590	M		
6	990	M		
11	PP_5062	M	[7]	
12	PP_A938	F		
13	PP_A942	F		
14	PP_A946	M		
15	PP_A983	M		
16	PP_A984	F		
17	PP_A985	M		
18	PP_A987	F		
19	PP_A988	M		
20	PP_A989	F		
5	898	M	[31]	
7	1097	F		
8	1452	F		
9	1581	F		
10	1852	F		
21	53	F	[6]	Sumatran *(P. abelii)*
26	550	F		
27	732	M		
29	1600	M		
30	PA_A953	F	[7]	
31	PA_A955	F		
32	PA_A964	F		
33	PA_B017	F		
34	PA_B018	M		
35	PA_B020	F		
22	154	F	[31]	
23	446	F		
24	498	M		
25	511	F		
28	1302	F		
36	695	F	[6]	Tapanuli *(P. tapanuliensis)*
37	PA_B019	M	[7]	

To identify ancestry informative markers (AIMs) distributed across the orang-utan genome, we split the master VCF by chromosome in R [40] and used the package adegenet 2.0 to calculate pairwise F_ST_ (fixation index) [41]. Because the number of SNPs needed to determine population structure is inversely proportionate to F_ST_ [42], sampling bias can impact F_ST_ values and thus affect selection of informative SNPs [43]. Consequently, to account for effects of stratification and minimize their impact on downstream association studies, populations with an F_ST_ < 0.01 require more than 20,000 SNPs for accurate inference, while upwards of 100,000 SNPs are needed for populations with an F_ST_ of 0.001 [44]. We therefore retained only the top 5000 biallelic SNPs per chromosome with the highest pairwise F_ST_ for each population; i.e. the number required to meet a goal of ~ 120,000 known AIMs. We then performed a PCA and DAPC in adegenet to confirm the SNPs’ utility in informing population structure.

We supplemented these with 51,128 additional SNP positions derived from 71 zoo-housed orang-utans that we genotyped from whole blood or tissue-derived DNAs on the Illumina iScan platform. We first extracted genomic DNA using either the Maxwell RSC Blood DNA or Tissue DNA kits, respectively, as automated on the Maxwell RSC instrument (Promega). We then used the Multi-Ethnic Global Array (MEGA) chip (Illumina), having used BLAST to compare the probes from each of the manufacturer’s commercial human microarrays to determine that MEGA had the highest proportion (61.27%) of total probes with single best hit (proportional to the total size of the manifest). We analysed the resulting IDAT files separately for each species in GenomeStudio 2.0 (Illumina). We first visualized sample performance by plotting the call rate against the P10 value; selected any samples that fell outside the majority cluster of samples; and excluded these poorly performing samples. After updating SNP statistics, we then filtered out SNPs based on low call quality: those that did not clearly cluster into heterozygotes and homozygotes (based on a Cluster Sep score < 0.3); those for which more than 10% lacked calls across samples; and those with an AB R Mean (mean of the normalized intensity – R – values for the AB genotypes) < 0.12. We again updated SNP statistics, re-clustered all remaining biallelic SNPs, and exported the resulting new cluster positions as a custom cluster file for downstream processing. We then filtered the custom cluster by minor allele frequency (MAF) > 0.01 and converted the final GenomeStudio file to VCF using the iScanVCFMerge tool (Fountain et al.*,* in review).

### Selection of Y-chromosomal targets

In the absence of a Y chromosome in the (female) orang-utan reference genome (ponAbe3), we designed probes for human (hg19) SNP positions that can be consistently successfully target-enriched in commercial human SeqCap panels. As numerous prior studies have successfully mapped male orang-utan sequences to the human Y-chromosome, we anticipated high on-target hybrid capture efficiency [31].

### Selection of medically relevant genes

We selected medically relevant genes in two ways. First, through a literature review, we prepared a list of genes either known or presumed to be pathogenic for cardiovascular and/or chronic respiratory diseases in humans, capitalizing on the genetic similarity of the human and orang-utan genomes. We then used the NCBI Gene database to search for each gene. The database calculates ortholog gene groups with the NCBI Eukaryotic Genome Annotation pipeline using protein sequence similarity and local synteny information. This process enabled us to view and search for documented orthologs within the orang-utan genome, and to determine their start and end positions. Second, we cross-referenced our list of genes with those previously identified by Roche Sequencing Solutions as potentially medically relevant, based on their inclusion in three SeqCap-based target-enrichment products: the SeqCap EZ MedExome panel, and the SeqCap EZ Share Prime Choice panels for Cardiomyopathy and for Channelopathy and Arrhythmias. For any genes in these panels not on our prior list, we principally used the UCSC Table Browser to derive exon positions for each gene on the orang-utan genome. For those not present in the Table Browser, we retrieved exon positions from the annotated Generic Feature Format (.GFF) file.

We complemented this set of genes with 33 additional genes identified by the American College of Medical Genetics and Genomics as being implicated in a variety of other human diseases, and which are recommended for reporting of secondary findings (SF v2.0) [45]. These might therefore be linked to health disorders or be indicators of in- and outbreeding depression in orang-utans. Their list includes 59 genes linked to conditions with definable clinical features, which have reliable clinical genetic tests that could facilitate early diagnosis, and which thus could lead to effective interventions or treatments. Because our aforementioned cardiac-relevant genes overlap with the ACMG SF v2.0, our panel in fact comprises all 59 genes as recommended by the ACMG.

### Proof-of-concept application of target-enrichment technology

We designed and synthesized probes using a commercial hybrid capture technology for target enrichment. A range of commercial products is available, and some have been previously used in non-human primates. However, the majority of all such studies to date have used off-the-shelf, mass-produced, pre-designed panels to enrich targets based on probes designed from the human genome, leading to high off-target coverage. ‘SureSelect’ technology (Agilent) has been used to enrich the exomes of chimpanzees (*Pan troglodytes*) and crab-eating *(Macaca fascicularis)*, Japanese *(M. fuscata)* and rhesus macaques *(M. mulatta)* (Human All Exon kits, [46, 47]), plus mitochondrial genomes in great apes [48]. Kits by Roche NimbleGen (SeqCap EZ Exome Probes 2.0) and Integrated DNA Technologies (xGen Exome Research Panel 1.0) have been used to capture and sequence whole exomes in both sifakas *(Propithecus verreauxi)* and *M. mulatta* [49]*.*

We instead chose to develop a custom panel based on ‘SeqCap’ target enrichment technology by Roche Sequencing Solutions, which evolved from the aforementioned Nimblegen technology. An earlier version by Nimblegen, the SeqCap EZ Developer Library, was previously successfully used to design custom exome enrichment probes around the chimpanzee reference genome [50]. In general, ‘SeqCap’ presents three major advantages over other commercial kits. First, it uses the Roche Universal Blocking Oligo (UBO), which reduces off-target sequencing by preventing library adapter sequences from annealing and being carried through the hybridization reaction. This applies Human COT DNA, rather than requiring a species-specific COT DNA, to mask repetitive elements. Second, Roche has published standardized ‘HyperPrep’ workflows for laboratory procedures, and pipelines for downstream data analysis that rely on open-source – versus commercial or proprietary – software tools (e.g. GATK [36]). Third, the entire laboratory workflow is performed in a single tube, reducing the potential for human and cross-contamination, and can accommodate either mechanical or enzymatic shearing.

To evaluate the utility of SeqCap technology in orang-utans, we first applied the SeqCap EZ MedExome panel – designed to target enrich the human exome, with higher coverage of medically relevant genes – to genomic DNA derived from nine orang-utans. We extracted genomic DNA from whole blood as aforementioned; applied the probes following the standard KAPA HyperPrep workflow (with mechanical shearing on a Covaris instrument); and multiplexed and sequenced the enriched targets at 50x coverage on an Illumina HiSeq 2500 paired-end rapid run. Mean sequence coverage was 55x with on-target enrichment of 89.2%, thus demonstrating SeqCap efficacy. We used the resulting sequence data as a reference when designing (or re-designing) probes around our custom orang-utan targets.

### Probe design for custom SeqCap panel

We designed a set of overlapping hybrid capture probes, ranging from 50 to 100 nt in length, around each target using Roche’s proprietary platforms. To prevent cross-hybridization to untargeted loci, we removed any probes containing 15-mers overrepresented in the ponAbe3 build. We then performed a pairwise analysis of the probe sequences against the ponAbe3 reference genome, using SSAHA [50], and selected probes with fewer than 21 potential matches to non-target sites elsewhere in the genome (90% identity over 30-mer subsequences). Probes targeting isolated SNPs were increased in concentration 2-fold to increase capture frequency and balance capture yields in relation to exon targets. To evaluate the utility of the loci for which probes could be designed, we re-genotyped the 37 whole genome sequences at all SNP-panel loci (as previously described) and pulled variants within the medically relevant gene regions by using SelectVariants in GATK on our recalibrated master VCF.

## Results

We present ponAbe3 genomic co-ordinates for 175,186 SNP loci, of which 124,060 were derived from our GATK analysis of published orang-utan whole-genome sequences and 51,126 from novel iScan genotyping of orang-utans. These include 165,344 autosomal SNPs, 9782 X-chromosome SNPs, 59 SNPs on unknown chromosomes, and 1 mitochondrial SNP. Of these, 1375 are located in exons. Co-ordinates, sources (i.e. GATK vs iScan), and gene information (i.e. transcript ID, exon number and ID, gene name; where applicable) are reported in the supporting document (SNP_Targets_ponAbe3_bed_file.txt). We further present 2315 hg19 Y-chromosomal targets spanning 0.167 Mb (ChrY_Targets_hg19_bed_file.txt). Of all these targets, SeqCap probes could be successfully designed for a total of 141,156 of the SNP loci (of which 1360 are in exons) and for all 2315 Y-chromosomal targets. Loci statistics per chromosome are presented in Table 2.

**Table 2:** Distribution of SNP panel loci, as computed in silico from the 37 re-sequenced whole genome sequences. Data are presented for all those loci in the panel, and again for only those loci for which SeqCap probes could be successfully designed. Further statistics can be found in the supplementary data (SNP_Targets_ponAbe3_bed_file.txt)

Location	Panel		SeqCap Probes	
	No. Loci	No. in Exons	No. Loci	No. in Exons
chr1	5344	107	4882	107
chr2A	4989	54	3946	54
chr2B	8403	65	6711	64
chr3	7120	72	5802	69
chr4	7090	56	5707	56
chr5	6948	55	5417	52
chr6	6655	59	5553	58
chr7	6024	58	4697	58
chr8	7199	47	5323	47
chr9	8111	66	6442	66
chr10	4992	66	4617	65
chr11	3566	50	3168	50
chr12	6049	60	4517	60
chr13	6695	22	4978	22
chr14	2835	35	2434	35
chr15	5829	46	4762	44
chr16	12,784	104	11,080	104
chr17	22,994	127	18,399	124
chr18	8720	22	7444	22
chr19	5976	71	4488	71
chr20	9660	50	8342	50
chr21	3610	27	3019	27
chr22	3751	32	3035	32
chrX	9782	16	6334	15
chrM	1	–	0	–
chrUn	59	8	59	8
**Total**	**175,186**	**1375**	**141,156**	**1360**

Of the medically relevant genes selected, we were able to design probes for 109 genes either known or suspected to be pathogenic for cardiac disease in humans; 43 genes either known or suspected to be pathogenic for respiratory diseases in humans; plus all 33 of the additional genes from the ACMG SF v2.0. Only two genes had sections that could not be covered by our probes: SDHD and BRCA1, which were unrepresented for 117 bp and 7 bp respectively. From the in-silico re-genotyping of each gene, we observed 1375 SNP loci within all exons. The supporting documents report a list of all genes, their associated disease and source, and the distribution of SNPs per gene (MedRel_Targets_ponAbe3.txt); in addition to the REF/ALT and MAF for each identified SNP (MedRel_Targets_REF_ALT_and_MAF_ponAbe3.txt).

Our final SeqCap panel size totalled 17.896 Mb, of which 17.045 Mb comprised the SNP and Y-chromosomal targets, and 0.851 Mb comprised the medically relevant genes.

## Discussion

Our targets are intended for use in three principal applications: building pedigrees; inferring ancestry; and for the study of genes potentially pathogenic for disease in orang-utans. As such, the resulting data can be ‘pruned’ to meet the diverse needs of downstream analyses. Researchers might identify kinship-informative SNPs in their populations by pruning for those with low linkage disequilibrium (LD) and high MAF, calculating their identity by descent (IBD), and comparing relatedness measures against known familial relationships. Ancestry could be inferred by downsizing the data to only AIMs, based on the sampled population’s F_ST_ values. Disease susceptibility and aetiology can be studied through comparison of known deleterious alleles in humans, and through linkage and quantitative trait loci (QTL) mapping, and genome-wide association study (GWAS) approaches. The power to do so is greatly increased when combined with phenotype data, and thus should be of particular value in studies of rehabilitant and captive (e.g. zoo) populations.

In the longer term, our panel could be expanded to include other valuable targets. We had considered adding genes from the Major Histocompatibility Complex (MHC), for example, given their critical involvement in immune response and pathogen defence. However, the MHC is characterised by allelic polymorphism, high gene density and copy number variation, which would greatly increase sequencing costs at the present time [51]. Further, preliminary studies in orang-utans have shown especially diverse and complicated MHC transcription profiles; previously unreported MHC class I alleles; and novel variation (among hominids) in gene copy number [52]. Designing targets based on so few available reference genomes, and so little published MHC data, could cause us to miss significant content and potentially misrepresent the true complexity of the region in our panel. More focused studies of the orang-utan MHC are thus needed to better define the target, in order to facilitate effective probe design. The panel might also be enhanced to include microsatellite loci, enabling ‘backwards compatibility’ with the volumes of microsatellite genotype data generated in the genus to date. At this time, however, the extensive repeats in these regions precluded our ability to design effective probes. It would therefore be better to apply our panel to samples previously genotyped at microsatellite loci. Developing technologies now render this achievable, even with the highly degraded and non-invasively produced samples that constitute the majority of orang-utan DNA collected to date: notably, fluorescence-activated cell-sorting (fecalFACS) has facilitated high-coverage, minimally biased sequencing of an entire mammalian genome from faeces [53]. Consequently, there is potential to re-analyze those samples with our panel to capitalize on the greater utility offered by SNPs. These are present at much greater density, provide better resolution for meiotic events, and offer more data for identifying some types of copy-number polymorphisms.

The extent to which targeted sequencing approaches can be broadly implemented to increase the efficiency, scope and impact of conservation genomic efforts will be dependent on the availability of cost-effective commercial products. The underlying technologies are rapidly evolving; thus, our use of the SeqCap product constitutes a minimum of what might be possible. At present, the feasibility of SeqCap with orang-utan targets is comparable to what can be achieved using off-the-shelf human-target-enrichment products, in that certain regions present technical challenges in both species. A prominent section of the orang-utan BRCA1 gene, for example, comprises a single repeat and corresponds to the same section of human BRCA1 that is similarly difficult to sequence and not often covered by human medical exome kits. As technology progresses, newer products can be expected to feature improved probe fidelity and target coverage, plus enhanced coverage uniformity and increased sequencing efficiency. Notably, Roche’s KAPA Target Enrichment product is scheduled for release in 2020; other potential products include xGen probe pools (Integrated DNA Technologies), Twist custom panels (TWIST Bioscience) and SureSelect (Agilent).

We estimate the cost savings of target enrichment to be substantial. The cost of sequencing a whole human genome at 30x coverage still averages $1000 in US laboratories, excluding the costs of sample and library preparation, genome mapping to a reference, annotating potentially clinically relevant variants, and storing the resulting data. In contrast, target enrichment pools can be multiplexed to increase sample capacity. In the case of SeqCap technology, dual- versus single-indexing can be used to increase multiplexing capacity, maintaining high sequencing coverage while avoiding excessive amounts of data from small target sizes [54]. Using SeqCap probes and single indexes, for example, our panel could be target-enriched and sequenced at 45x coverage in up to 16 orang-utans, in a single lane of an Illumina MiSeq v2 run, at a sequencing cost of $1812 ($113.25 per sample). Utilizing dual indexing, we could achieve the same sequencing coverage on an Illumina HiSeq4000 at a cost of $2819 for 192 samples ($14.68 per sample – a significant cost saving). As SeqCap technology has already been successfully applied to non-invasive (i.e. faecal) samples [55], the utility of our probes could also expand to studies of natural populations.

## Conclusions

This panel has now been standardized for use in *The Orang-utan Conservation Genetics Project*, a global effort to study the genetics of wild, ex-captive and zoo-housed orang-utans. More than 3200 DNA samples have been collected globally from orang-utans to date. Using the SeqCap technology described herein, we are enriching and sequencing this panel of targets in ~ 1000 individual orang-utans. We encourage other researchers to adopt this panel to facilitate comparative studies of orang-utan population genomics. The panel is compatible with a range of commercial target-enrichment products, can be synthesized in whole or in part, and may be multiplexed and scaled for large sample sizes at low cost.

## Supplementary Information

**Additional file 1.** SNP_Targets_ponAbe3_bed_file. Bed file for SNP targets, sources and locations.

**Additional file 2.** ChrY_Targets_hg19. Bed file for Y-chromosomal targets.

**Additional file 3.** MedRel_Targets_ponAbe3. List of medically relevant genes in the panel.

**Additional file 4 **MedRel_Targets_REF_ALT_and_MAF_ponAbe3. Statistics for SNP loci called in-silico in medically relevant genes.

## Data Availability

The co-ordinates of all targets identified in this study are published with this manuscript as supplementary text files, in which the fourth and fifth columns (where applicable) indicate the source from which the target was identified and whether or not probes could be designed for the target using SeqCap technology. The first through third column in each file can be extracted and saved in .bed format for downstream use. Restrictions apply to the availability of raw microarray and sequence data that derived from biomaterials licensed only for this study. These data may be available from the corresponding author upon reasonable request and with the permission of each licensor.

## References

[1] Bruce EJ, Ayala FJ (1978). Humans and apes are genetically very similar. Nature..

[2] Lucotte G, Smith DG (1982). Distinction électrophorétique entre les deux sous-espéces d’Orang-outan. Hum Genet.

[3] Xu X, Arnason U (1996). The mitochondrial DNA molecule of Sumatran orangutan and a molecular proposal for two (Bornean and Sumatran) species of orangutan. J Mol Evol.

[4] Warren KS, Verschoor EJ, Langenhuijzen S, Heriyanto SRA, Vigilant L (2001). Speciation and intrasubspecific variation of Bornean orangutans, *Pongo pygmaeus pygmaeus*. Mol Biol Evol.

[5] Singleton I, Wich S, Husson S, Stephens S, Atmoko SU, Leighton M, et al. Orangutan population and habitat viability assessment: final report: IUCN/SSC Conservation Breeding Specialist Group; 2004.

[6] Locke DP, Hillier LW, Warren WC, Worley KC, Nazareth LV, Muzny DM (2011). Comparative and demographic analysis of orang-utan genomes. Nature..

[7] Nater A, Mattle-Greminger MP, Nurcahyo A, Nowak MG, de Manuel M, Desai T (2017). Morphometric, behavioral, and genomic evidence for a new orangutan species. Curr Biol.

[8] Voigt M, Wich SA, Ancrenaz M, Meijaard E, Abram N, Banes GL (2018). Global demand for natural resources eliminated more than 100,000 Bornean Orangutans. Curr Biol.

[9] Utami-Atmoko S, Traylor-Holzer K, Rifqi MA, et al. Orangutan population and habitat viability assessment: final report: IUCN/SSC Conservation Breeding Specialist Group; 2019.

[10] Arora N, Nater A, van Schaik CP, Willems EP, van Noordwijk MA, Goossens B (2010). Effects of Pleistocene glaciations and rivers on the population structure of Bornean orangutans *(Pongo pygmaeus)*. Proc Natl Acad Sci.

[11] Banes GL, Galdikas BMF, Vigilant L (2016). Reintroduction of confiscated and displaced mammals risks outbreeding and introgression in natural populations, as evidenced by orang-utans of divergent subspecies. Sci Rep.

[12] Banes G, Chua W, Elder M, Kao J (2018). Orang-utans *Pongo* spp in Asian zoos: current status, challenges and progress towards long-term population sustainability. Int Zoo Yearb.

[13] Galdikas BMF, Wood JW (1990). Birth spacing patterns in humans and apes. Am J Phys Anthropol.

[14] Lowenstine LJ, McManamon R, Bonar C. Preliminary results of a survey of United States and Canadian orangutan mortalities in the north American SSP population from 1980–March 2008. Proc Amer Assoc Zoo Vet. 2008;40.

[15] Smith J, Lung N. Results of the 2012 U.S. Orangutan health survey. Proc Amer Assoc Zoo Vet. 2012.

[16] Murphy HW, Danforth MD, Clyde VL (2018). The great ape heart project. Int Zoo Yearb.

[17] Strong V, Martin M, Redrobe S, White K, Baiker K (2018). A retrospective review of great ape cardiovascular disease epidemiology and pathology. Int Zoo Yearb.

[18] Palmer A. Ethical debates in orangutan conservation: Routledge; 2020.

[19] Rietkerk F, Pereboom JJM (2018). Editorial: conservation of great apes. Int Zoo Yearb.

[20] Zhang Y, Ryder OA, Zhang Y (2001). Genetic divergence of orangutan subspecies (*Pongo pygmaeus*). J Mol Evol.

[21] Warren KS, Nijmian IJ, Lenstra JA, Swan RA, Heriyanto, den Boer M (2000). Microsatellite DNA variation in Bornean orangutans (*Pongo pygmaeus*). J Med Primatol.

[22] Goossens B, Chikhi L, Jalil MF, Ancrenaz M, Lackman-Ancrenaz I, Mohamed M, Andau P, Bruford MW (2005). Patterns of genetic diversity and migration in increasingly fragmented and declining orang-utan (*Pongo pygmaeus*) populations from Sabah, Malaysia. Mol Ecol.

[23] Goossens B, Setchell JM, James SS, Funk SM, Chikhi L, Abulani A, Ancrenaz M, Lackman-Ancrenaz I, Bruford MW (2006). Philopatry and reproductive success in Bornean orang-utans *(Pongo pygmaeus)*. Mol Ecol.

[24] Kanthaswamy S, Kurushima JD, Smith DG (2006). Inferring *Pongo* conservation units: a perspective based on microsatellite and mitochondrial DNA analyses. Primates..

[25] Morrogh-Bernard HC, Morf NV, Chivers DJ, Krützen M (2010). Dispersal patterns of orang-utans (*Pongo* spp.) in a Bornean peat-swamp forest. Int J Primatol.

[26] Nietlisbach P, Arora N, Nater A, Goossens B, Van Schaik CP, Krützen M (2012). Heavily male-biased long-distance dispersal of orang-utans (genus: *Pongo*), as revealed by Y-chromosomal and mitochondrial genetic markers. Mol Ecol.

[27] Nater A, Arora N, Greminger MP, van Schaik CP, Singleton I, Wich SA (2013). Marked population structure and recent migration in the critically endangered Sumatran orangutan (*Pongo abelii*). J Hered.

[28] Banes GL, Galdikas BMF, Vigilant L (2015). Male orang-utan bimaturism and reproductive success at Camp Leakey in Tanjung Puting National Park, Indonesia. Behav Ecol Sociobiol.

[29] Tajima T, Malim TP, Inoue E (2018). Reproductive success of two male morphs in a free-ranging population of Bornean orangutans. Primates..

[30] Städele V, Vigilant L (2016). Strategies for determining kinship in wild populations using genetic data. Ecol Evol.

[31] Prado-Martinez J, Sudmant PH, Kidd JM, Li H, Kelley JL, Lorente-Galdos B (2013). Great ape genetic diversity and population history. Nature..

[32] Kronenberg ZN, Fiddes IT, Gordon D, Murali S, Cantsilieris S, Meyerson OS (2018). High-resolution comparative analysis of great ape genomes. Science.

[33] Li H. Aligning sequence reads, clone sequences and assembly contigs with BWA-MEM. arXiv. 2013:1303.3997v2.

[34] Li H, Handsaker B, Wysoker A, Fennell T, Ruan J, Homer N (2009). The sequence alignment/map format and SAMtools. Bioinform Oxf Engl.

[35] Broad Institute. Picard Tools. http://broadinstitute.github.io/picard/.

[36] Poplin R, Ruano-Rubio V, DePristo MA, Fennell TJ, Carneiro MO, van der Auwera GA, et al. Scaling accurate genetic variant discovery to tens of thousands of samples. Biorxiv. 2018:201178.

[37] der Auwera GAV, Carneiro MO, Hartl C, Poplin R, Angel GD, Levy-Moonshine A (2013). From FastQ data to high confidence variant calls: the genome analysis toolkit best practices pipeline. Curr Protoc Bioinformatics.

[38] Thain D, Tannenbaum T, Livny M (2005). Distributed computing in practice: the condor experience. Concurrency Comput Pract Exp.

[39] Weitzel D, Zvada M, Vukotic I, Gardner RW, Bockelman BP (2019). StashCache: a distributed caching federation for the open science grid. PEARC ‘19.

[40] R Core Team. R: A language and environment for statistical computing. R Foundation for Statistical Computing, Vienna, Austria. http://www.R-project.org/. Accessed 1 Mar 2020.

[41] Jombart T, Ahmed I (2011). adegenet 1.3-1: new tools for the analysis of genome-wide SNP data. Bioinform Oxf Engl.

[42] Patterson N, Price AL, Reich D (2006). Population structure and eigenanalysis. PLoS Genet.

[43] Henriques D, Parejo M, Vignal A, Wragg D, Wallberg A, Webster MT (2018). Developing reduced SNP assays from whole-genome sequence data to estimate introgression in an organism with complex genetic patterns, the Iberian honeybee *(Apis mellifera iberiensis)*. Evol Appl.

[44] Price AL, Patterson NJ, Plenge RM, Weinblatt ME, Shadick NA, Reich D (2006). Principal components analysis corrects for stratification in genome-wide association studies. Nat Genet.

[45] Kalia SS, Adelman K, Bale SJ, Chung WK, Eng C, Evans JP (2016). Recommendations for reporting of secondary findings in clinical exome and genome sequencing, 2016 update (ACMG SF v2.0): a policy statement of the American College of Medical Genetics and Genomics. Genet Med.

[46] Jin X, He M, Ferguson B, Meng Y, Ouyang L, Ren J (2012). An effort to use human-based exome capture methods to analyze chimpanzee and macaque exomes. PLoS One.

[47] Vallender EJ (2011). Expanding whole exome resequencing into non-human primates. Genome Biol.

[48] Hallast P, Delser P, Batini C, Zadik D, Rocchi M, Schempp W (2016). Great ape Y chromosome and mitochondrial DNA phylogenies reflect subspecies structure and patterns of mating and dispersal. Genome Res.

[49] Webster TH, Guevara EE, Lawler RR, Bradley BJ. Successful exome capture and sequencing in lemurs using human baits. bioRxiv. 2018:490839.

[50] Hernandez-Rodriguez J, Arandjelovic M, Lester J, de Filippo C, Weihmann A, Meyer M (2017). The impact of endogenous content, replicates and pooling on genome capture from fecal samples. Mol Ecol Resour.

[51] Ning Z, Cox AJ, Mullikin JC (2001). SSAHA: a fast search method for large DNA databases. Genome Res.

[52] de Groot NG, Otting N, Maccari G, Robinson J, Hammond JA, Blancher A (2019). Nomenclature report 2019: major histocompatibility complex genes and alleles of great and small ape and old and new world monkey species. Immunogenetics..

[53] de Groot NG, Heijmans CMC, van der Wiel MKH, Blokhuis JH, Mulder A, Guethlein LA (2016). Complex MHC class I gene transcription profiles and their functional impact in orangutans. J Immunol.

[54] Orkin JD, Montague MJ, Tejada-Martinez D, de Manuel M, del Campo J, et al. Selection and local adaptation in capuchin monkeys revealed through fluorescence-activated cell sorting of feces (fecalFACS). bioRxiv. 2020:366112.

[55] van der Werf IM, Kooy RF, Vandeweyer G (2015). A robust protocol to increase NimbleGen SeqCap EZ multiplexing capacity to 96 samples. PLoS One.

